# Unravelling the role of contrasting light environments in shaping leaf functional traits: a global meta-analysis

**DOI:** 10.3389/fpls.2026.1884395

**Published:** 2026-07-14

**Authors:** Wajee ul Hassan, Xiping Cheng, Yanfang Wang, Runze Li, Pengyue Dai, Jing Chen

**Affiliations:** 1 College of Forestry Southwest Forestry University Kunming, Yunnan, China; 2 College of Soil and Water Conservation, Southwest Forestry University, Kunming, Yunnan, China; 3 College of Gardening and Horticulture, Southwest Forestry University, Kunming, Yunnan, China

**Keywords:** leaf economics spectrum, leaf mass area, meta-analysis, natural light gradient, photosynthetic rates, plant functional group, SLA, specific leaf area

## Abstract

Leaf functional traits play a decisive role in harnessing plant performance and the functionality of leaf functional traits is largely governed by change in light environments yet at global scale this mechanistic relationship of leaf functional traits with light under diverse plant communities and abiotic conditions is still poorly understood due to conflicting findings across regions. To quantify this relationship globally, we aimed to verify the effects of contrasting light environments on leaf functional traits worldwide in (I) different plant community factors; plant functional group, leaf form and leaf habit (II) different abiotic variables, that is, mean annual precipitation (MAP), mean annual temperature (MAT), elevation and climate; and (III) verify whether leaf functional traits exhibit variations in different regions across the globe in contrasting light environments. we conducted a meta-analysis of worldwide experimental data from 1980–2024 including 536 pairwise comparisons to evaluate the influence of different light environments on leaf functional traits both morphological such as specific leaf area, stomatal density, leaf mass area and physiological leaf traits such as net photosynthetic rate, chlorophyll content, leaf dry matter content. Our results showed that leaf morphological traits significantly responded to contrasting light environments and these effects were more pronounced in areas with moderate climatic conditions (MAT 5>15 °C and >500 mm of MAP) and species with evergreen leaves. Leaf physiological functional traits showed more significant sensitivity to moderate to high temperature (MAT >15 °C) and deciduous leaf habit along with regional variations due to integrating effects of MAP and MAT. However, negligible support was found for leaf dry matter content. Our work provides trait-level plastic responses along natural light gradient, modulated by climate and plant community factors and provide an insight into leaf functional traits adaptive strategies across light gradient worldwide.

## 1 Introduction

Light is considered to be the key heterogenous environmental determinant that shapes the plant growth and morphology as intensity changes (Pearcy, 2007; Yang et al., 2018). All plants have the apparatus to capture the incoming light and transmute it into chemical energy this process of photosynthesis not only assists plant life progression but also caters to the energy for the extended food webs in the hierarchy of the ecosystem (Niyogi and Wolosiuk, 2015). This echoes the paramount importance of light use efficiency and its driving responses in forest ecosystem. Scientists have consistently supported the notion that different abiotic and plant community factors such as the natural characteristics of species and climate factors are responsible for ecological zoning of plants species creating a stellar biodiversity in forest ecosystem (García-Palacios et al., 2018). However, solar radiation has been the most significant limiting factor for species composition and forests proliferation (Jagodziński et al., 2016; Tang and Dubayah, 2017; Perot et al., 2017; Chou et al., 2018).

Light intensity, for instance is known to cause myriad alterations in morphological and physiological variations along with manipulating the photosynthetic apparatus of the leaves (Givnish, 1988; Muraoka et al., 1997; Niinemets, 1997; Vieira et al., 2011). Fluctuation in light intensity is bound to regulate the integration of transcription and photoreceptors and drive photosynthetic activity hence directly influencing the plant growth and development (Vieira et al., 2011; Song et al., 2019). All of these findings support the understanding of plant species response to changing light environment and predict changes in future climate patterns. Understanding the surface level response of plant to changing light environment is crucial but what is more important is how plant respond to it down to traits level, and that involves the understanding of leaf functional traits, The leaf functional traits such as specific leaf area, stomatal density, Leaf mass area and leaf dry matter (LDMC) content are decisive factors in evaluating plant adaptation to varying light environments (Guo et al., 2019). The effects of light on leaf functional traits are far reaching since these functional traits are pivotal in deciding plant ecological strategies and highly influenced by the environmental variations and settles the key assumption that morphological traits determine physiological performance, individual fitness and evolution of life history of the species (Ackerly and Monson, 2003; Violle et al., 2007; Adler et al., 2014). Over the course of the last few decades, the term trait has transcended from common life word to more scientifically in various disciplines and it is described as any measurable morpho-physiological and phenological feature of a plant, which is addressed by the potential of those features to impact plant fitness in the natural environment, ecosystem functioning and individual organismal performance (McIntyre et al., 1999; Geber and Griffen, 2003; Reich et al., 2003; Mcgill et al., 2006; Violle et al., 2007).

Specific leaf area (SLA) is a major trait in the worldwide leaf economics spectrum (Wright et al., 2004), and undergoes substantial changes in response to light variations. An abundant amount of research has been carried out and reported the mechanistic effect of changing light environment on Leaf functional traits especially Specific leaf area. Plants in low light environments possess thinner leaves and lower leaf biomass per unit area consequently augmenting light interception per unit of leaf biomass. However, higher SLA in low light environment also results in low epidermis production making leaves vulnerable to photoinhibition in the future (Pearcy, 2007; Valladares and Niinemets, 2007; Gratani, 2014). High light intensity (Niinemets et al., 2015) can cause plants to develop low specific leaf area and strategize against adverse climatic conditions. This is a classic example of ambivalent effects of a key functional trait in a changing light environment. Changes in SLA imply that it helps in plant performance under varying light conditions (Gratani, 2014). However, the functionality of this trait is not linear, instead it is supposedly influenced by the climatic and geographical factor. For instance, in a study carried out in North-American tropical forest, the change in light environment had no significant effects on SLA, conversely SLA was fairly affected by the changing light environment in European temperate forests and subtropical deciduous forest species (Guzmán and Cordero, 2013; Puglielli et al., 2017; Granata et al., 2020). This supports the notion that the plasticity of functional traits is highly influenced by geographic and species- environment interactions; therefore, our meta-analysis seeks to address the inconsistencies in leaf trait variation among different regional and ecological gradients to better understand the response of functional traits to a contrasting light environment.

Recent evidence shows that, while the light environment profoundly affects the specific leaf area (Matos et al., 2009; Zhang et al., 2022) and leaf mass area of evergreen species in the Mediterranean climate (Gratani et al., 2006) also prominently affects stomatal density. For example, stomatal density increased under high light intensity in deciduous species in the subtropical region of Europe (Granata et al., 2020). Besides light affecting leaf morphological traits, physiological leaf functional traits also undergo plasticity as light changes. Recent evidence shows that changes in the light environment profoundly alters the chlorophyll content (Barbosa-Campos et al., 2018) and net photosynthetic rate (Pn) (Zhang et al., 2022). The chlorophyll content of the leaves is associated with the interaction of photosystems responsible for absorbing light at different wavelengths and the change in photosystem stoichiometry can optimize the electron transport and ameliorate the unbalanced absorption of light by the two photosystems thereby improving the photosynthetic efficiency (Chow et al., 1990; Walters, 2004).

We found that the previously documented evidence marked variation in leaf functional traits and by combining the leaf functional traits with abiotic and plant community moderators our meta-analysis proposes the central hypothesis that in forest ecosystem, do chief leaf functional traits such as SLA, LMA, stomatal density, chlorophyll content, Pn, and LDMC respond differently to high light as compared to low light when testing under different abiotic and plant community moderators across the globe. Ultimately this meta-analysis would help us understand the functionality of leaf traits at a global scale in contrasting light environments and we will be able to verify whether changes in the light environment affect leaf functional traits in (I) different plant community variables; plant functional group, leaf form and leaf habit (II) different abiotic variables, that is, MAT, MAP, elevation and climate; and (III) verify whether different regions show variation in leaf functional traits in contrasting light environments.

## 2 Materials and methods

### 2.1 Literature search and selection of studies

**Figure 1:**
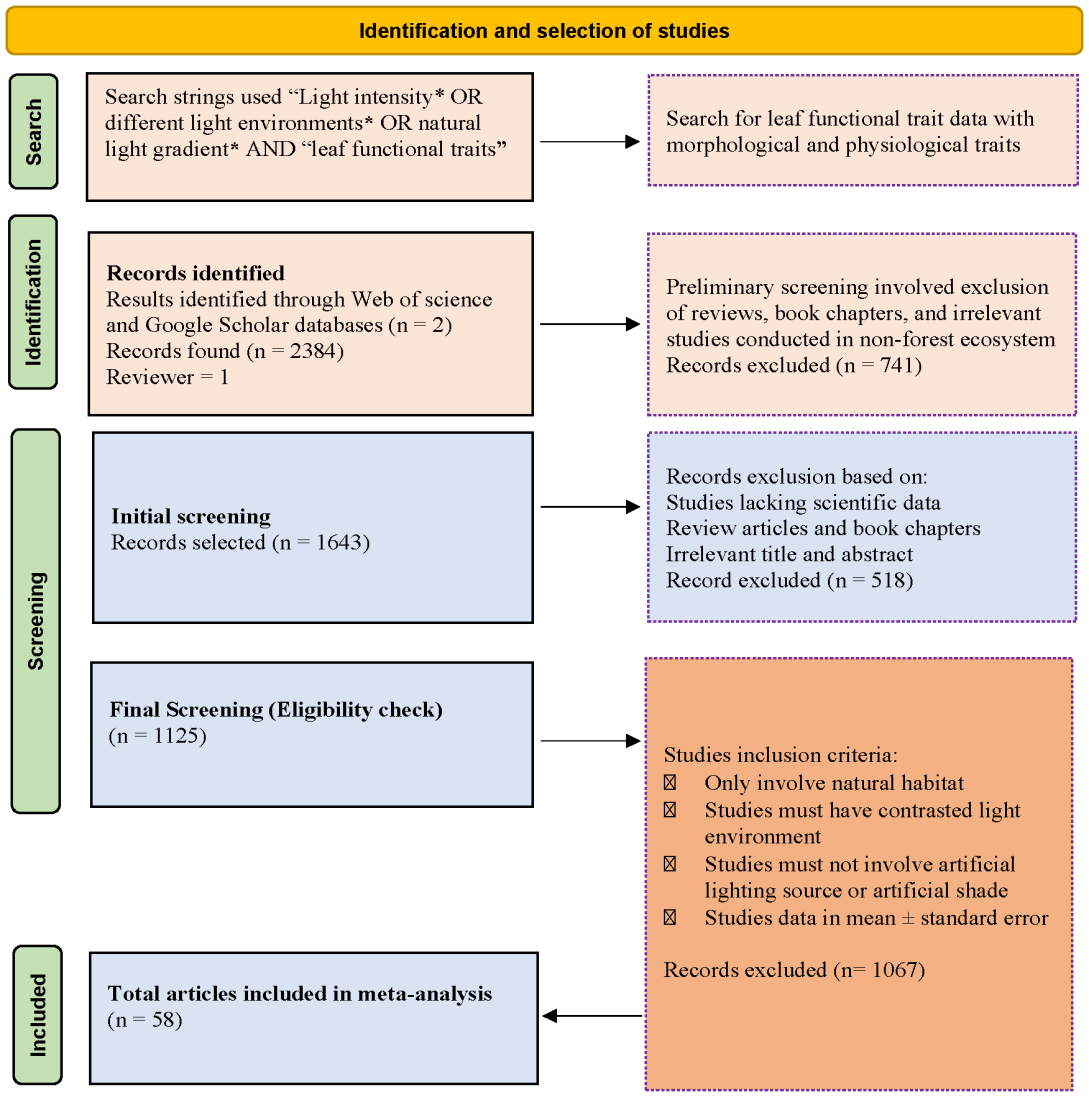
PRISMA (preferred reporting items for systematic reviews and meta-analysis) flow diagram of the meta-analysis. “*” symbol represent keywords used in the search string.

**Figure 2:**
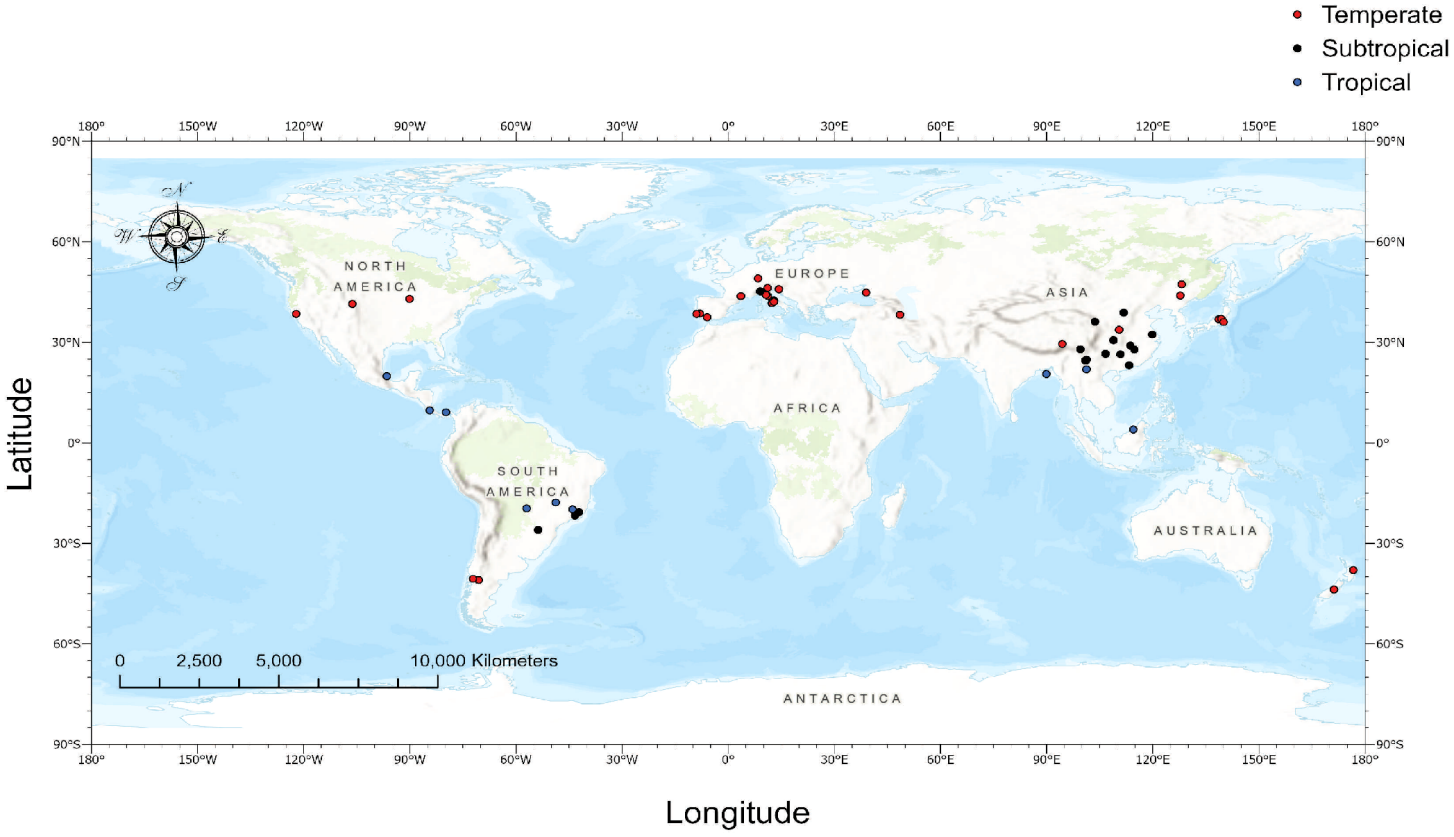
This map shows the geographic distribution of the study locations included in this meta-analysis. Each distribution point represents a study location (total number of locations: n = 58). The map was created in ArcGIS Pro using a standard basemap.

Our meta-analysis involved the steps outlined by the Preferred Reporting Items for Systematic Reviews and Meta-analyses (PRISMA). To outline a meta-analysis, the steps taken for study selection and data extraction must be clearly defined and described. These steps followed a documented and reproducible protocol (Liberati et al., 2009). A flowchart with steps taken according to the PRISMA guidelines of our meta-analysis is shown in the (**Figure 1**). On 22 August, 2025, we conducted a search for published literature in the Web of Science and Google Scholar database using the search strings “light intensity* OR different light environments* OR natural light gradient* AND leaf functional traits” also shown in (**Figure 1**) retrieving 2384 articles. After excluding review articles, book chapters, and other reports lacking scientific data and involving non-forest ecosystem, we selected 1125 articles for final screening. We established the following inclusion criteria for selecting studies ultimately included in the meta-analysis: (1) studies must be from natural light habitats; (2) studies must present contrasting lighting environments (high and low light); (3) experiments must not involve artificial light sources or artificial shades; and (4) studies must provide extractable mean ± standard error (SE) data. This screening resulted in 58 studies with 536 comparisons that were included in our meta-analysis. A list of the data sources along with the data is available in data availability section. The geographic distribution of the study locations included in this meta-analysis is represented in the map (**Figure 2**).

### 2.2 Data retrieval, categorization and calculation of response ratios (RRs)

Many authors directly reported the data in the text of the papers. Some authors presented the values in the form of tabulated data, which were extracted directly and integrated as mean (X), standard deviation (SD), and sample size (n) for the control and experimental groups, respectively. If the relevant data were only presented in the plots, we contacted the corresponding authors and asked for numerical values. If the authors did not respond, the data were retrieved from figures in the articles using the WebPlotDigitizer tool (https://automeris.io/) version 5.2 (Rohatgi, 2024).

If any of the response variables are expressed as standard error (SE), the following formula is used for its conversion to standard deviation using python (v.3.14.0).


$$SD=SE\sqrt{n}$$

Where, SD represents the standard deviation, SE represents the standard error, and n represents the sample size. We included the following response variables: specific leaf area (SLA), leaf mass area (LMA), stomatal density, chlorophyll content and net photosynthetic rate (Pn). A brief description of the response variables is provided in the (**Table 1**). To investigate the influence of abiotic and plant community moderators on response variables we extracted the data of climate, mean annual temperature, mean annual precipitation and elevation, and the plant community characteristics including; plant functional group, leaf habit, and leaf form from studies. If the original published literature did not mention the bioclimatic variables of the study site, we extracted the corresponding bioclimatic variables using the worldclim dataset (Fick and Hijmans, 2017) (https://www.worldclim.org) with the spatial resolution of 30 seconds. We compared the response variables between contrasting light environments then used moderating variables, we classified moderating variables into following categories; Region (Asia, Europe, Australia, South America, North America), Plant functional group (Tree, Shrub, Herb), MAP (0-500, >500), MAT (0-5 °C -, 5-15 °C, >15 °C), Leaf habit (Evergreen, Deciduous), leaf form (Simple, Compound), Elevation (0-1000, 1000-3500) and climate (Temperate, Subtropical, Tropical).

**Table 1:** Brief description of the response variables used in the meta-analysis.

Response variables	Abbreviation	Units	Functional significance
Specific leaf area	SLA	cm2 g−1	Light capture, plant growth rate and ecological strategy (Reich et al., 1992; Wright et al., 2004)
Leaf mass per area	LMA	g m-2	A measure of investment in structure, provide support for carbon gain and longevity (Wright et al., 2004)
Stomatal density	SD	mm-2	Responsible for leaf gas exchange (Poorter et al., 2025)
Net photosynthetic rate	Pn	µmol m-2 s-1	Organic matter accumulation and indicator for photosynthetic capacity (Wu et al., 2023)
Chlorophyll content	CC	mg g-1	photosynthesis activity, mutations, stress and nutritional state (Niinemets and Tenhunen, 1997; Wu et al., 2008)
Leaf dry matter content	LDMC	Mg g-1	Resistance to drought and leaf litter decomposition (Yang et al., 2019)

Response ratios have been used in ecological meta-analysis to calculate the impact of treatments on response variables and to better quantify the relationships between variables (Dieleman et al., 2010). Our meta-analysis calculated leaf functional trait responses to light availability and evaluate the relative changes of these response variables. The natural logarithm of the response ratio (RR) was used as the effect sizes as described previously (Hedges et al., 1999). The RR equation is as follows:


$$RR=\ln R\left(\frac{{\bar{x}}_{t}}{{\bar{x}}_{c}}\right)=\ln\left({\bar{x}}_{t}\right)-\ln\left({\bar{x}}_{c}\right)$$

In the above formula, ${\bar{x}}_{t}$ represents the mean values of the response variable in the treatment group (high light), whereas ${\bar{x}}_{c}$ represents the control group (low light). The variance (*v*) is calculated using the following formula:


$$\text{v}=\frac{s_{t}^{2}}{n_{t}{\bar{x}}_{t}^{2}}+\frac{s_{c}^{2}}{n_{c}{\bar{x}}_{c}^{2}}$$

Where *s* represents the standard deviation (SD), $\bar{x}$ is the standard mean and *n* is the sample size for each response variable in the treatment group (*t*) or in the control group (*c*).


$$\text{w}=\frac{1}{\text{v}}$$

The non-parametric weight factor (w) was used to weight the results for all effect sizes.


$$\ln RR_{++}=\frac{\sum \ln R_{i}\times w_{i}}{\sum w_{i}}$$

SDlnRR++= 1∑wi

In the above formulas $\ln{\text{RR}}_{++}$ and ${\text{SD}}_{\ln RR++}$ represent the weighted average effect size and variance after non-parametric weighting, respectively.

This study calculated the 95% confidence intervals (CIs) of effect sizes using a random effects model. We employed the inverse variance statistical method and a random effects model because they are generally used for studying level differences as compared to the fixed effect model which is suitable only when there is a sample level difference between studies (Chen et al., 2012). Finally, we extracted the RR_++_ values ​​and bootstrap confidence intervals for each variable in the contrasting light environments. The significance level of the effect of light intensity using moderators on the response variable was estimated by the null hypothesis implying that if the confidence interval overlaps the zero line, which means that contrasting light environments do not have a significant effect on leaf functional traits. If the confidence interval does not overlap the zero line it means that contrasting light environments have a significant effect on the response variables.

Publication bias and heterogeneity tests:

First, the publication bias was assessed using the Rosenthal fail-safe N approach (**Supplementary Table 1**). Then, we used the I^2^ and T^2^ tests to estimate heterogeneity **Supplementary Figure 1**. The value of I^2^ from 0–100 percentage and T^2^ scores show low to high heterogeneity. The following equation was used for I^2^ test:


$$I^{2}=\left(Q-\;\frac{df}{Q}\right)\times 100$$

T^2^ was calculated using the following equation:


$$T^{2}=\frac{Q-df}{C}$$

### 2.3 Statistical analysis

Statistical analysis of this meta-analysis was performed using the R program (version. 4.1), and openmee software was used to perform the meta-analysis. In addition to the statistical tests explained above, we evaluated the impact of moderators on leaf functional traitsusing permutation based random forest (RF) analysis by implementing the `rfpermute´ package in R program. The RF was fitted with 500 trees (ntree = 500) and mtry= 2. This analysis was used to evaluate the moderators in terms of their importance in determining the response of leaf functional traits towards contrasting light environments. The percentage importance of the mean squared error %IncMSE was extracted and used as an indicator to measure the importance of each moderating variable. The performance of the model was then explained using the % var explained R^2^ from a random forest using the moderating variables. Graphics were generated and gathered in graphpad prism 10 (GraphPad Software, Boston, USA).

## 3 Results

### 3.1 Overall summary of the meta-analysis

This meta-analysis included 58 studies comprising 536 pairwise comparisons of leaf functional traits in contrasting light environments. The experiments were conducted in temperate, tropical and subtropical biomes of the Asia, Europe, Australia, North America and South America. The findings revealed that the higher variations in leaf morphological functional traits were caused by plant functional group, MAT and MAP, while functional traits related to leaf physiology showed higher variations in plant functional group and leaf habit.

### 3.2 Light profoundly influences the leaf morphological functional traits

**Figure 3:**
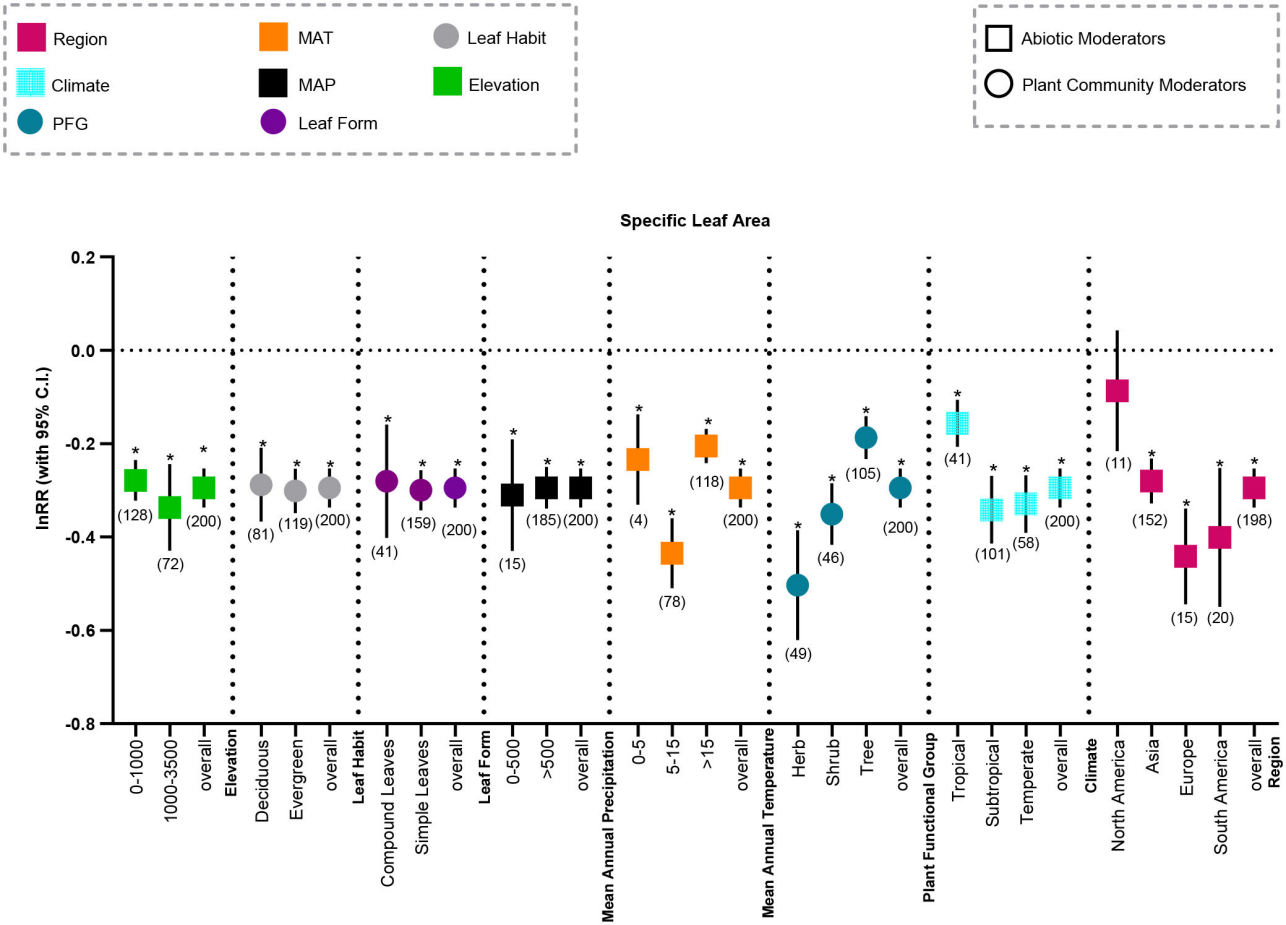
Subgroup analysis of specific leaf area in response to contrasting light environments. The effect is considered significant if the 95% confidence interval line does not intersect the zero-value indicated with horizontal dotted line (p<0.05). The p value is displayed for the moderating variables using asterisks *(p < 0.05). The numbers at the bottom of the CI represent the sample size.

Our results justified the use of meta-analytic and random forest model. The overall effects of contrasting light environment on SLAwere significant (p<0.05) (**Figure 3**). Regarding the abiotic moderators, SLA responded more significantly to light intensity in South America (95% CI: -0.550 to -0.252), Asia (95% CI: -0.328 to -0.232) and Europe (95% CI: -0.544 to -0.339) (p< 0.05) (**Figure 3**). The climate and elevation caused myriad variations in SLA as it was profoundly affected by light intensity in temperate (95% CI: -0.391 to -0.268), subtropical (95% CI: -0.414 to -0.269) and tropical climates (95% CI: -0.207 to -0.106) (p< 0.05); similar significant pattern was observed in elevation moderators as SLA exhibited significant responses to an elevation range of 1000-3500 (95% CI: -0.429 to -0.244) and 0-1000m range (95% CI: -0.322 to -0.235) (p< 0.05) (**Figure 3**). Precipitation and temperature play crucial roles in causing plasticity in leaf functional traits, SLA showed profound variations in all mean annual precipitation ranges (MAP) with more significant effects in 0–500 mm (95% CI: -0.430 to -0.191) (p< 0.05) and exhibited variations among all mean annual temperature (MAT) ranges with more pronounced effects in 5-15 °C temperature range (95% CI: -0.510 to -0.360) (p value< 0.05) (**Figure 3**). Regarding plant community moderators, SLA showed profound variations in plant functional group with great significant variations in herb (95% CI: -0.621 to -0.386) (p< 0.05) (**Figure 3**). The leaf form also largely determined the patterns of SLA to light intensity, SLA showed profound response to light intensity in both simple (95% CI: -0.343 to -0.257) and compound leaves (95% CI: -0.402 to -0.159) (p< 0.05) (**Figure 3**), and a similar trend was observed in leaf habit as SLA showed a significant response to light intensity in both evergreen (95% CI: -0.349 to -0.254) and deciduous leaves (95% CI: -0.367 to -0.209) (p< 0.05) (**Figure 3**). However, such variation was not evident for all moderating variables. For example, SLA showed no significant variation in light intensity in North America.

**Figure 4:**
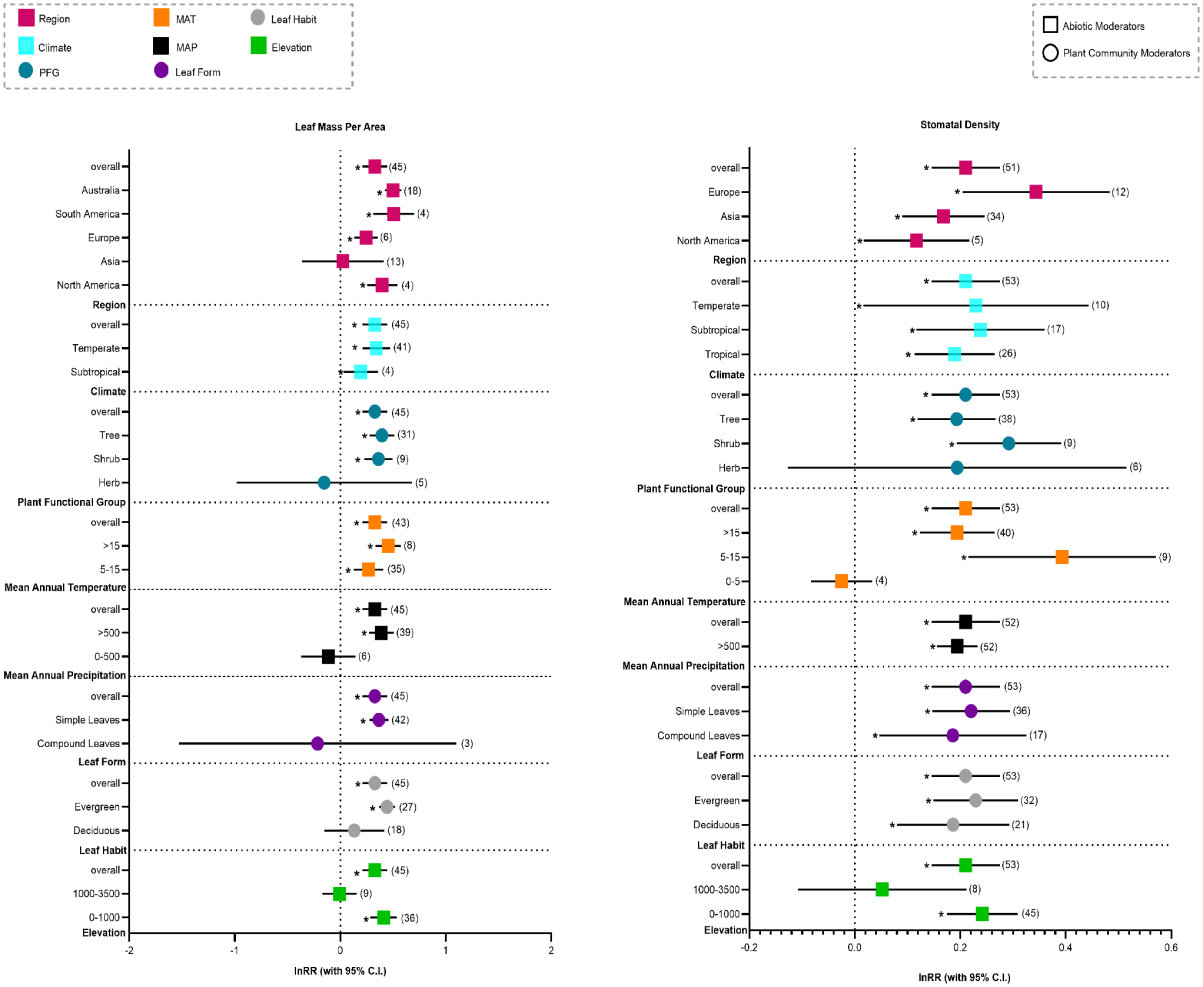
Subgroup analysis of leaf mass per area and stomatal density in response to contrasting light environments. The effect is considered significant if the 95% confidence interval line does not intersect the zero-value indicated with vertical dotted line (p<0.05). The p value is displayed for the moderating variables using asterisks *(p < 0.05). The sample size is represented by numbers on the right of the CI.

We also found significant overall effects on leaf mass area (LMA) and stomatal density (p<0.05) (**Figure 4**). Regarding abiotic moderators, LMA and stomatal density showed profound variations in all regions (p< 0.05) except for LMA in Asia and stomatal density in South America (**Figure 4**). Similarly, LMA and stomatal density were significantly affected by light intensity in all climate and elevation ranges (p< 0.05) except for the elevation range of 1000–3500 m (**Figure 4**). LMA and stomatal density showed great variation in areas with precipitation range of >500 mm (p< 0.05) and areas with mean annual temperature (MAT) of 5-15 °C and >15 °C (p< 0.05) (**Figure 4**). Regarding the plant community moderators, LMA and stomatal density showed profound variations in plant functional group (p< 0.05) except herb (**Figure 4**), While Leaf habit and leaf form favored the response in both LMA and stomatal density in all moderators (p< 0.05) except for LMA, which showed no significant response in deciduous leaves and compound leaves (**Figure 4**). These results echo the importance of spatial disparity and its moderating effects on leaf morphological functional traits at various light levels. Some moderating variables not included in the meta-analysis due to too low sample size (threshold ≥3).

### 3.3 Natural light gradient profoundly influences the leaf physiological functional traits

**Figure 5:**
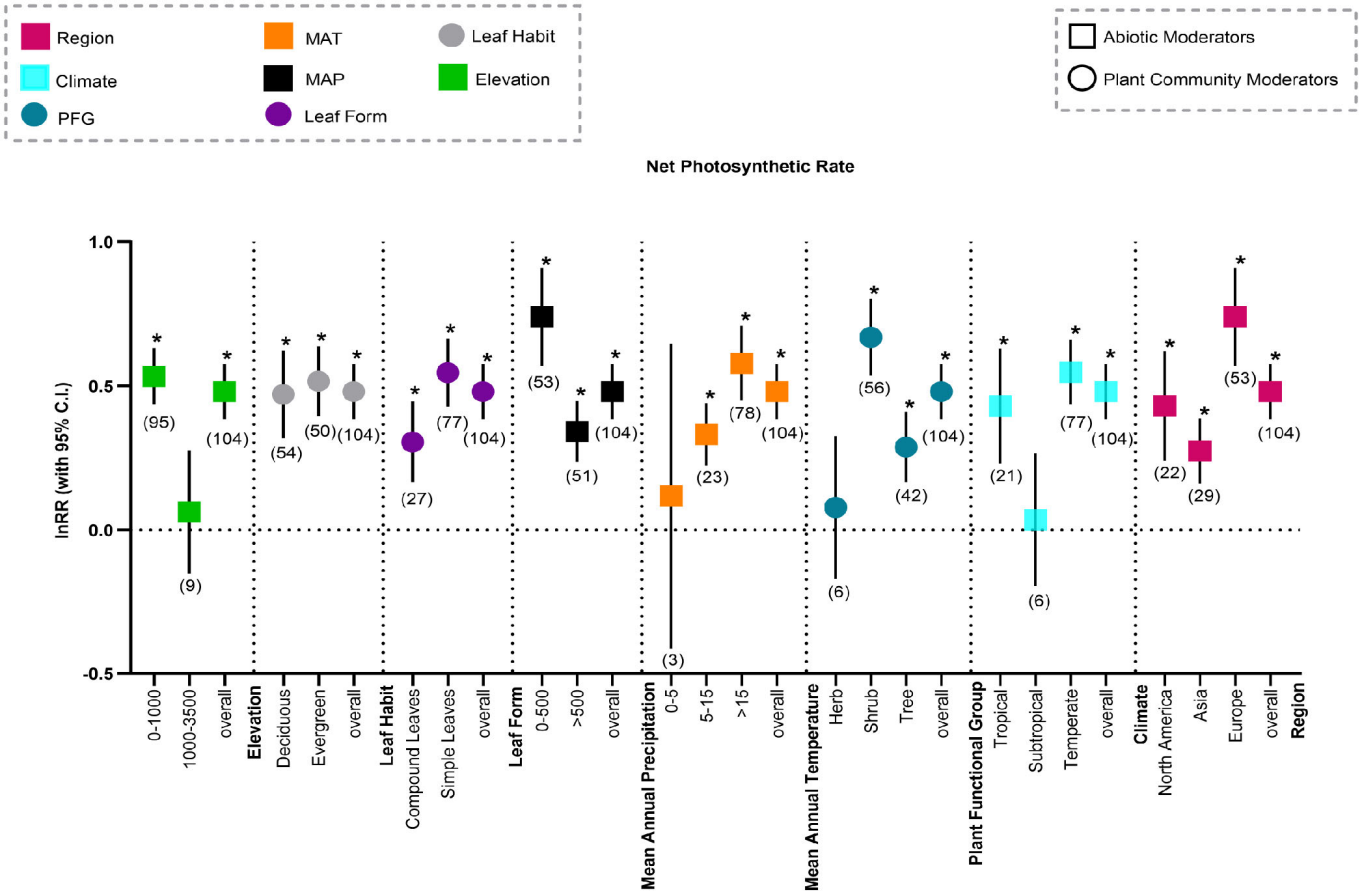
Subgroup analysis of net photosynthetic rate in response to contrasting light environments. The effect is considered significant if the 95% confidence interval line does not intersect the zero-value indicated with horizontal dotted line (p<0.05). The p value is displayed for the moderating variables using asterisks *(p < 0.05). The sample size is represented by numbers at the bottom of the CI.

The overall effects of contrasting light environments on the Pn were significant for the abiotic and plant community moderators (p<0.05) (**Figure 5**). Regarding the abiotic moderators. Pnshowed significant variations to high light intensity in all regions (p< 0.05) (**Figure 5**), The climate and elevation moderators caused great variations in Pn as it was profoundly affected by light intensity in temperate (95% CI: 0.434 to 0.659) and tropical climates (95% CI: 0.229 to 0.629) (p< 0.05) yet no significant variation was evident in the subtropical climate (**Figure 5**), Pn showed a greater response to light gradient in the 0-1000m elevation range (95% CI: 0.434 to 0.632) (p< 0.05) (**Figure 5**). The Pn was considerably affected overall in all MAP ranges and the same trend was observed in MAT (p< 0.05) (**Figure 5**). Regarding the plant community moderators, Pn was significantly influenced by high light intensity among all plant functional group (p< 0.05) except for herb (**Figure 5**). The leaf forms and leaf habit also largely determined the patterns of leaf functional traits to light gradient; Pn showed a profound response to light intensity in all leaf forms and leaf habit moderators (p< 0.05) (**Figure 5**).

**Figure 6:**
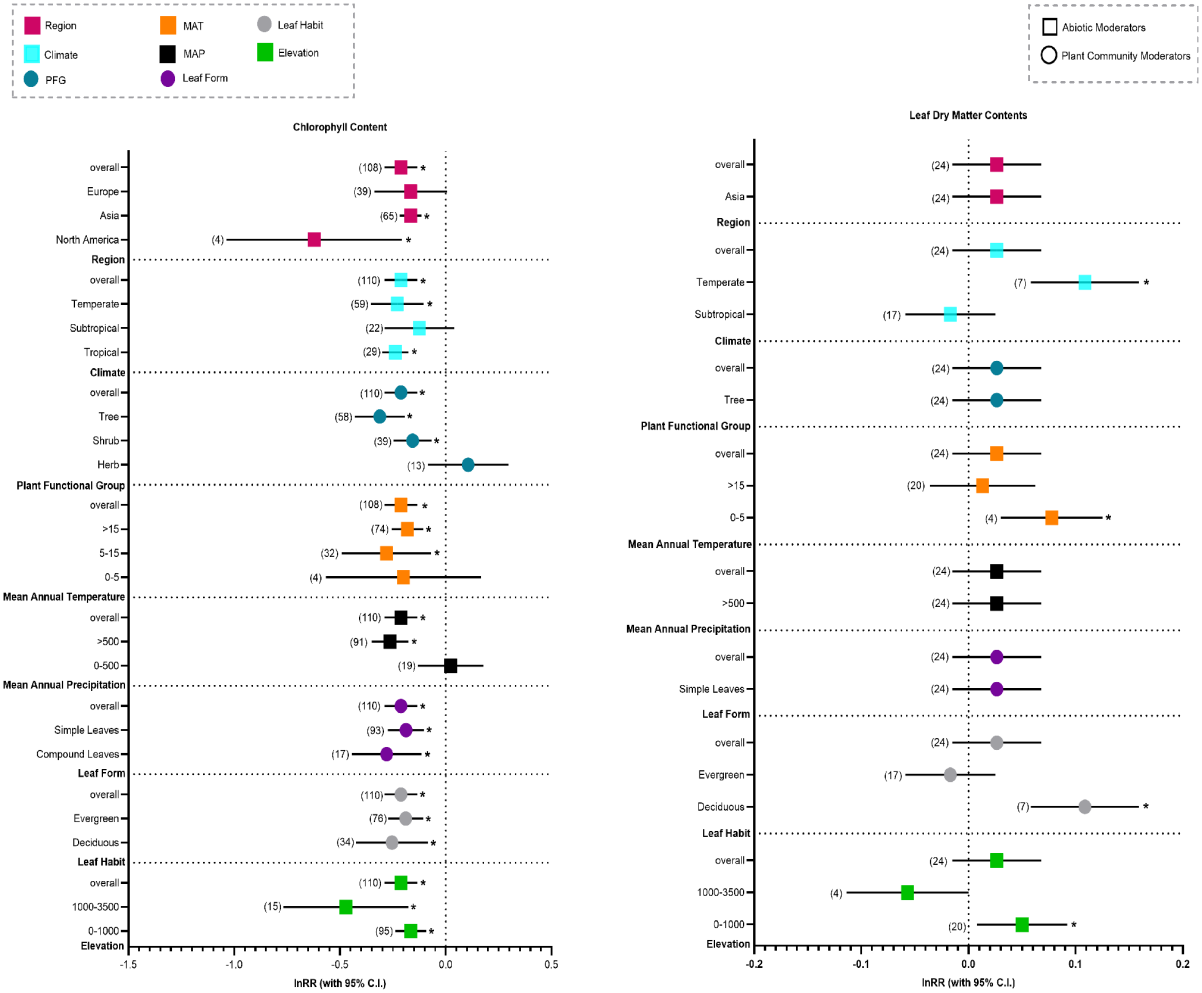
Subgroup analysis of chlorophyll content and leaf dry matter content in response to contrasting light environments. The effect is considered significant if the 95% confidence interval line does not intersect the zero-value indicated with vertical dotted line (p<0.05). The p value is displayed for the moderating variables using asterisks: *(p < 0.05). The numbers in the parenthesis on the left of the CI represent the sample size.

We found overall significant variations in chlorophyll content to region moderators (p< 0.05), except that no variations were observed in chlorophyll content in Europe and South America. (**Figure 6**). However, the LDMC exhibited no significant variations in any region. Furthermore, chlorophyll content a showed profound response to light intensity in all climate and elevation ranges (p< 0.05) except the subtropical climate (**Figure 6**), whereas, LDMC failed to show an overall significant variation in climate and elevation despite a positive response in the elevation range of 0–1000 m and temperate climate (**Figure 6**). The MAT and MAP moderating variables favored the variation in chlorophyll content (p< 0.05) whereas LDMC showed overall no significant variation in MAT and MAP moderators (**Figure 6**). Regarding the plant community moderators, chlorophyll content exhibited overall profound variations in plant functional group, leaf habit and leaf form moderating variables (p< 0.05) except that no significant response was evident in herb PFG (**Figure 6**), LDMC on the other hand did not significantly differ between all moderators except marginally significant variations in deciduous leaf habit (p< 0.05) (**Figure 6**).

### 3.4 Moderating effects of plant community and abiotic variables on leaf functional traits

**Figure 7:**
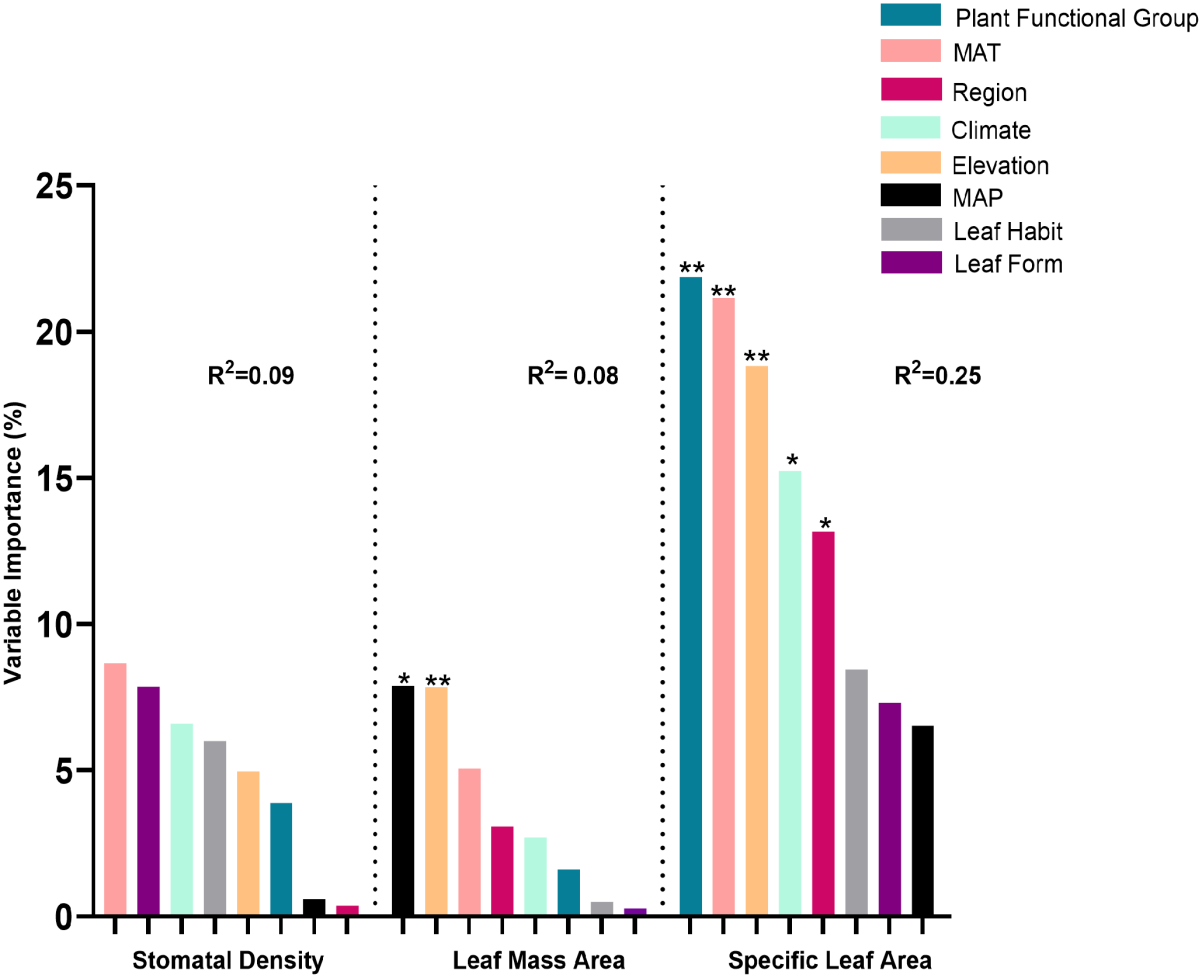
Effect of moderators on to the Specific leaf area, Leaf mass area, Stomatal density under the influence of contrasting light environments. The legend colors represent different moderating variables; The statistical significance is represented with asterisk (*)p< 0.05; (**)p*<* 0.01. p< 0.05 shows a significant effect of abiotic and plant community moderators on leaf functional traits, while p< 0.01 indicates a highly profound effect. The established range for R^2^ is 0 to 1 and it shows the strength and significance of the model. As the range of R^2^ increase, the explanatory power of the model correspondingly increases, respectively. MAT is the mean annual temperature; MAP is the mean annual precipitation.

In our meta-analysis, we computed lnRR effect sizes of leaf functional traits and observed the effects of abiotic and plant community moderators. The analysis revealed that among all moderating variables, plant functional group (21.86%) and mean annual temperature (21.15%) were the strongest predictors and showed the highest percentage of variable importance in SLA followed by other predictors ordered by their percentage of variable importance such as region and climate (**Figure 7**). LMA was strongly influenced by the mean annual precipitation (7.88%) and elevation (7.84%) (**Figure 7**). The variable importance of stomatal density was strongly explained by mean annual temperature (8.66%) and leaf form (7.86%) with other moderating variables ranked by their percentage of variable importance (**Figure 7**).

**Figure 8:**
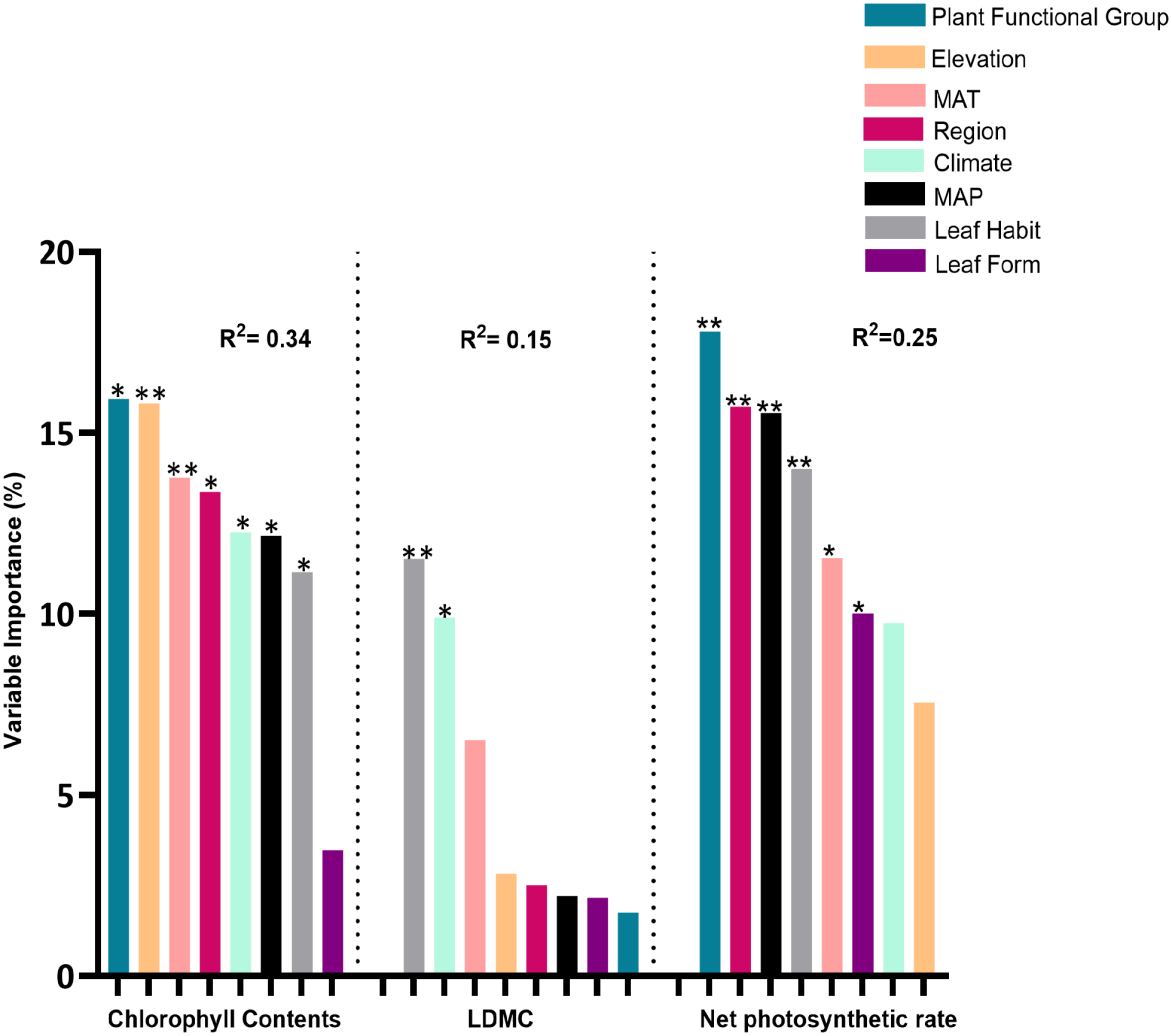
Effect of moderators on to the percentage variance of net photosynthetic rate, chlorophyll content and leaf dry matter content under the influence of contrasting lights. The legend colors represent different moderating variables; The statistical significance is represented with asterisk (*)p< 0.05; (**)p*<* 0.01. p< 0.05 indicates a significant effect of abiotic and plant community moderators on leaf functional traits, while p< 0.01 indicates a highly profound effect. The established range for R^2^ is 0 to 1 and it shows the strength and significance of the model. As the range of R^2^ increase, the explanatory power of the model correspondingly increases, respectively. LDMC is the leaf dry matter content; MAT is mean annual temperature; MAP is mean annual precipitation.

**Figure 9:**
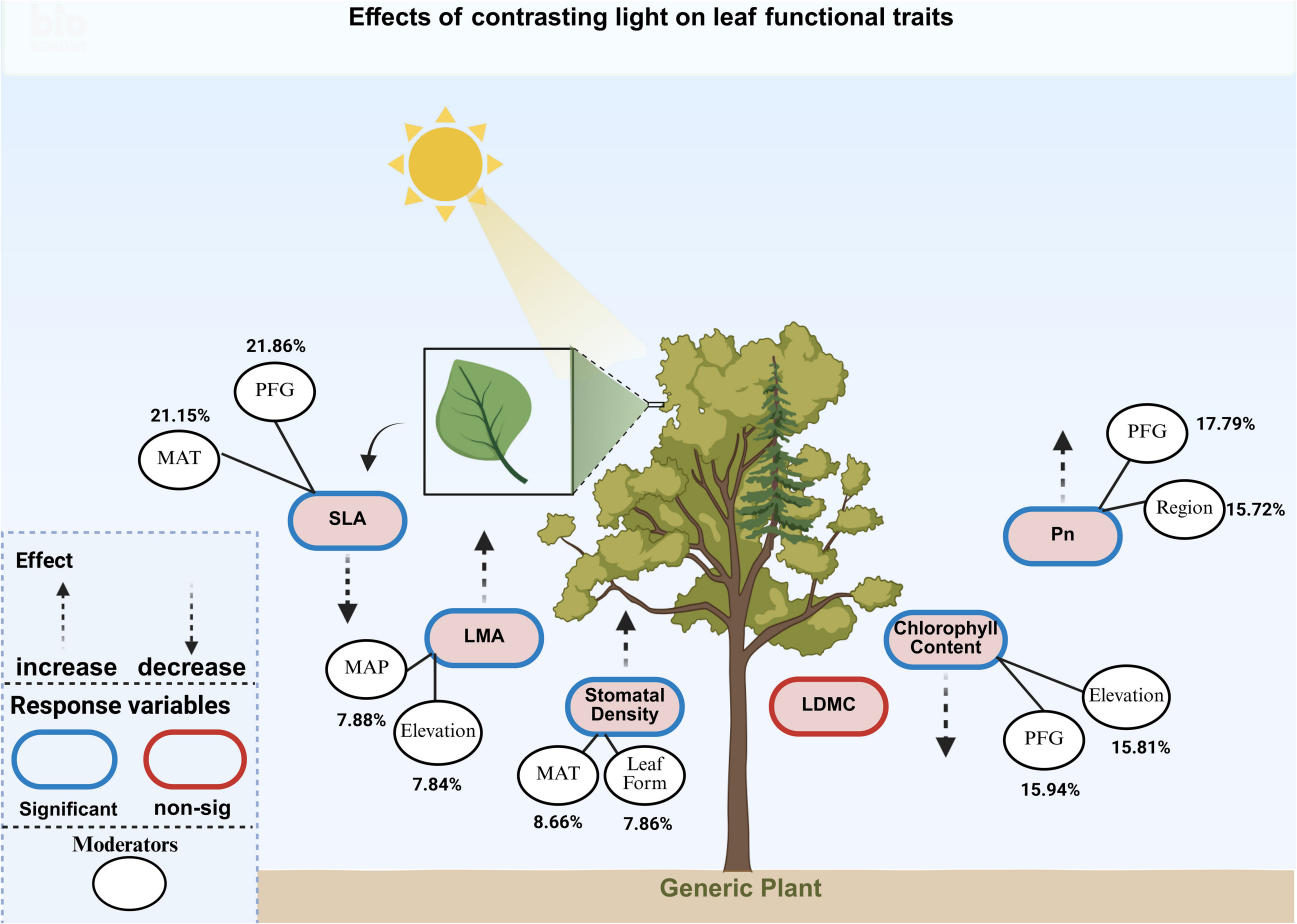
Overview diagram of the key results found in this meta-analysis, The increasing or decreasing effect of leaf functional traits under light is represented by arrowhead up and arrowhead down, respectively. The significantly affected response variables are represented with blue pills and non-significant response variable is shown by red pill. The top moderators for each response variables are represented by oval with their percentage of variable importance. SLA; specific leaf area; LMA, leaf mass area; LDMC, leaf dry matter content; Pn, net photosynthetic rate; MAP, mean annual precipitation; MAT, mean annual temperature; PFG, plant functional group. The figure was created using the BioRender and adobe illustrator. Created in BioRender. Hassan (2026) (https://BioRender.com/9ju0s2f).

Further analysis revealed that the, plant functional group (17.79%) and region (16.15%) had the highest percentage of variable importance in Pn followed by other moderators ordered by their percentage of variable importance such as mean annual precipitation and leaf habit (**Figure 8**). Conversely, chlorophyll content was mainly influenced by plant functional group (15.94%) and elevation (15.81%) along with other predictors ranked by their percentage of variable importance such as MAT and region (**Figure 8**). The percentage variance of LDMC was mainly explained by leaf habit (11.52%) and climate (9.90%) with other moderating variables ranked by their percentage variance explained, such as mean annual temperature and elevation (**Figure 8**). Overview diagram of the key results found in this meta-analysis are presented in (**Figure 9**).

## 4 Discussion

The goal of our global meta-analysis was to quantify the relationship between leaf functional traits and the natural light gradient and to investigate the influence of abiotic and plant community moderators on those key leaf functional traits across a global scale. While previous studies provide evidence about the effects of changes in the light environment on leaf functional traits, the association between the functionality of leaf traits and the variation brought about by abiotic and plant community factors across the globe was not evident. There are mechanisms that make all possible causal pathways viable: different plant functional group can lead to change in leaf phenological characteristics; leaf phenology can lead to changes in the behaviour of functional traits to increasing light, and behavioural changes can lead to heterogeneity among leaf functional traits across different regions. our analysis found evidence of the latter, that plant community and abiotic moderators influence how leaf functional traits respond to increasing light intensity and that there is a strong relationship between light and leaf functional traits.

In our meta-analysis, leaf functional traits responded differently to abiotic and plant community moderators under the influence of natural light gradient, and our results of decreased specific leaf area (SLA) among different moderating variables indicate the defensive mechanism of plants to adapt to a high light environment. Our findings revealed that among abiotic and plant community moderating variables, Plant functional groups (PFG) and mean annual temperature (MAT) contributed more profoundly to variations in SLA (**Figure 3**), which was also explained by the percentage of variable importance of these moderators (**Figure 7**). SLA showed profound variations in herb between plant functional group (**Figure 3**) possibly because herbs even though they have smaller effect sizes in this meta-analysis generally show more variation in SLA in contrasting light environments than trees and shrubs because of their flexible morphological structure. One possible explanation for this is that herbs have short life cycle and flexible structure (Klimeš et al., 2021) which allows them to develop faster and aid in better adaptation to temporal indicators as compared to woody plants as their development is a long-term process constrained by long-term goals and they have to invest much more in structural reinforcement than herbs (Poorter et al., 2012) hence woody plants have to make compromises when exploiting short-term heterogeneity such as the distribution of light gaps (Alméras and Fournier, 2009). This is consistent with the previous studies where contrasting light environments caused significant variation in SLA among different plant functional group such as trees (Hu et al., 2016) shrubs (Puglielli et al., 2017) and herbs (Tucić et al., 1998). These results show the pattern and adaptiveness of different plant functional group to high light and highlights the important underlying mechanism observed in studies over the years. Where plants grown under high light conditions typically develop thicker leaves and a smaller SLA partly due to a greater number of palisade tissue layers or longer cells. This increases the number of chloroplasts and the content of photosynthetic enzymes, thus improving the photosynthetic capacity per unit of leaf area (Hanson, 1917; Björkman, 1981). We analyzed and found in our meta-analysis that SLA showed profound variations in regions where the mean annual temperature ranged between 5 and 15 °C (**Figure 3**). One could explain why species in moderate to high temperatures produce thicker and lower SLA leaves by augmenting the layers of spongy or palisade mesophyll (Oguchi et al., 2003) and the leaves with lower SLA leads to the accumulation of heat-resistant proteins thus inducing adaptive evolution to environmental stress (Ackerly et al., 2002).

Our study expands the understanding of SLA in contrasting light habitats using leaf habit as moderator, and the response of SLA to contrasting light habitats was overall significant in leaf habit but showed a more profound response in evergreen leaves (**Figure 3**). This aligns with the two previous global analysis of leaf traits (Reich et al., 1997; Wright et al., 2005) which showed that evergreen species exhibited higher variation in specific leaf area (SLA) ​​than deciduous species. This could be due to (Xu et al., 2024) the longevity of evergreen trees as they inherently retain their green leaves all year round and capture light outside the main growing season leading them to produce more thicker and more efficient leaves under varying light condition, (Kikuzawa and Lechowicz, 2011) a result of somewhat compensating the higher structural reinforcement per unit dry mass. A previous study (Barbero et al., 1992) and empirical evidence (Gratani et al., 2006) demonstrated the greater ability of the evergreen oak *Quercus ilex* to adapt to both high and low light which explains the broader ecological distribution of evergreen species irrespective of the global change (Gratani et al., 2006). Findings from both individual and global synthesis demonstrated the potential of integrate leaf habit as a moderating variable in our meta-analysis. The increased variation of SLA at higher elevation range under the influence of light gradient in our results (**Figure 3**) could be due to the fact that plants (Zhang et al., 2024) which grow at high altitudes have developed phenotypic plasticity as a result of being exposed to strong ultraviolet radiations and harsh environments. For instance, evidence from studies of different plant functional group (PFG) showed greater variation in SLA at high elevation (Scheepens et al., 2010) (Woodward, 1983), and high elevations pose adverse stresses of low temperature and high radiation (Zhang et al., 2024), resulting in adaptive changes as a result of increased plasticity to extreme settings. Thus helping them cope with the higher altitude conditions (Zhang et al., 2021) and providing a possible explanation for the topographic heterogeneity in regulating the variation in SLA in our meta-analysis. The findings involving light interactions with SLA using moderators support the need for future experiments to further address interactions with SLA in large-scale manipulations.

Different environmental conditions can exert different selection pressures on plants, leading to a certain degree of trait differentiation. Leaf mass per unit area (LMA) and stomatal density are key functional traits that are closely related to the balance between resource availability and leaf lifespan providing a good central role for the leaf economic spectrum (Wright et al., 2004). Based on our findings, LMA varied profoundly in regions where the mean annual temperature range was 5-15 °C and >15 °C (**Figure 4**), which aligns with species showing significant variation in LMA in areas with high radiation and extreme climatic conditions (Niinemets, 2001; Wright et al., 2004). This variation in LMA is caused by the increase in leaf thickness due to increased temperature and high light (Niinemets, 2001) and interdependence of leaf density with LMA (De La Riva et al., 2016) leading to synergistically profound variations in LMA in regions with high temperature. Moreover, we also found a significant interaction between stomatal density and areas with increased temperature (**Figure 4**) with MAT being the strongest predictor of variance in stomatal density in contrasting light environments (**Figure 7**) in accord with the previous studies (Yan et al., 2017; Beerling, 1993). This could be because the rise in annual mean temperature over the last couple of decades has led to greater photosynthetic enzyme activity (Hill et al., 2015) and cell differentiation (Luomala et al., 2005), which may have caused plants to increase their stomatal density to maximize their photosynthetic rate establishing a functional role for this trait in a global climate change. Furthermore, the response of LMA in contrasting light habitats was more pronounced in evergreen leaves than in deciduous leaves (**Figure 4**) a potential explanation is the habit of evergreen leaves (Poorter et al., 2009) as they are inherently programmed to have a longer leaf life span and more structural reinforcement resulting in an increased variation in LMA, which is in accordance with the previous studies (Miyaji et al., 1997;  Poorter, 2001) where the variations are more pronounced in evergreen leaves exposed to high light environments as they exhibit higher LMA. On top of that, LMA showed significant variation among plant functional groups more profoundly in trees against contrasting light environments (**Figure 4**). This is in accordance with recent studies (Castro-Díez et al., 2000; Poorter et al., 2009) as the increase in LMA is likely driven by the increasing number of cells (Castro-Díez et al., 2000) and the slow expansion rate (Avalos et al., 1999) as leaf expansion rates are slower in species with low SLA (LMA = 1/SLA). Ultimately, leading to more variations in trees (Moles and Westoby, 2000), these observations also implicitly substantiate the slightly less variation in SLA in trees than herbs (**Figure 3**).

The differences in chlorophyll content between species may represent the integrating effect of species-specific response and differences in plasticity and this difference is associated with adaptation to the availability of light (Li et al., 2018). Our meta-analysis indicated an overall significant reduction in chlorophyll content to contrasting light environments, with more profound variations in trees and shrubs (**Figure 6**) these results are further explained by variable importance of plant functional group by being the top predictor in random forest analysis, this could be explained by the fact that high light can tempt shrubs to reduce chlorophyll synthesis in their leaves leading to a self-protection mechanism to avoid photoinhibition (Zhi-Zhen et al., 2014) and also cause a reduction in chlorophyll content in trees (La Porta et al., 2006) despite the inherent protection of photosynthetic pigments in line with studies where trees and shrubs showed significant reduction to high light in a natural light habitat. However, regarding the plant community moderators LDMC showed a significantly increasing response in deciduous leaves despite an overall insignificant response in leaf habit (**Figure 6**). such result could be because deciduous leaves exhibit fast growth (Fonseca et al., 2000) due to their resource acquisition strategy and short growing period, which maximizes resource capture (Poorter et al., 2009) and deciduous leaves with high LDMC tend to defend against tissue damage in leaf structure under extreme stresses (Freschet et al., 2010) Thus they are more likely to show more variations in deciduous leaves. Regarding the abiotic moderators, LDMC showed a profound sensitivity to high light in areas with mean annual temperature of 0-5 °C (**Figure 6**) which may be explained by plant species in cold environments tends to develop denser leaves with high LDMC (Stevenson, 2015) to avoid the risk of frost damage. This may be because prolonged exposure of plants to cold leads to accumulation of cold-resistant leaf functional traits, thus promoting a strong adaptive evolutionary traits (Körner, 1999). However, chlorophyll content showed profound sensitivity to light intensity in areas with slightly warming temperature (**Figure 6**) because (Nagata et al., 2005) chlorophyll synthesis requires a series of enzymatic reactions and warming will inhibit these enzymatic reactions and cause reduction in overall leaf chlorophyll content, Regarding the Pn as a response variable, it showed an overall increasing response to abiotic and plant community moderators (**Figure 5**) also explained by percentage of variable importance (**Figure 8**) with much more profound response in areas with temperatures above 15 °C (**Figure 5**). This may be explained by species in warm environments increase their threshold temperature (Berry and Bjorkman, 1980) above which warming cause inactivation of the photosynthetic temperatures by increasing the heat tolerance of photosynthetic apparatus and increasing the efficiency of electron transport, thus allowing the species to perform higher rate of photosynthesis at higher temperatures, which aligns with previous studies (Strain et al., 1976). Overall, the findings of our meta-analysis are consistent with the global framework of the leaf economic spectrum (LES), which posits that leaf traits are distributed between resource acquisition and conservation strategies. One side of the spectrum represents resource acquisition strategies are, characterized by a high leaf mass per area (LMA), high photosynthetic rate, and deciduous leaf habit of the species. Conversely, the other end represents resource conservation strategies characterized by low specific leaf area (SLA), high leaf dry matter content (LDMC), and evergreen leaf habit. This spectrum reflects the trade-offs and adaptive strategies of plants under various environmental conditions. Light, temperature, precipitation, and elevation collectively drive the functional strategies of plants. Specifically, low temperatures promote an increase in leaf dry matter content (LDMC), thereby reinforcing resource conservation strategies. Simultaneously, adjustments in SLA and LMA by evergreen leaves aid in adapting to light stress, our meta-analysis supports the global economic spectrum of leaves and clarifies the impacts of changes in abiotic and plant community factors on the leaf functional traits to light across the globe and provides a valuable insight for researchers about functionality of leaf traits and sustainable use of plant resources. While our findings reflect variations in leaf functional traits to changing light environment, the future studies could incorporate phylogenetic comparative methods to confirm that the adaptation of leaf functional traits is due to change in light environment rather than non-independence of the species.

## 5 Conclusion

This meta-analysis provides a new perspective on the behaviour of leaf functional traits in contrasting light environments globally; and the central idea of this study was derived from the plant-environment interaction to better understand the adaptive mechanisms of leaf functional traits to light environments under diverse abiotic and plant community conditions to cope with future climate changes. We found that PFG, leaf habit and hydrothermal conditions were the primary drivers of functional traits variations. Our meta-analysis further revealed that leaf morphological traits showed pronounced adaptive adjustments to contrasting light environments in moderate climatic conditions and evergreen leaf habit with compound leaf forms. while, leaf physiological traits exhibited significant variations under moderate thermal conditions except for LDMC. This study thoroughly elucidates the synergistic patterns of variation in leaf functional traits in response to contrasting light environments worldwide. These findings of our meta-analysis provide trait-level plastic responses along natural light gradient, modulated by climate and plant community factors and provide an insight into leaf functional traits adaptive strategies across light gradient.

## Data Availability

Publicly available datasets were analyzed in this study. This data can be found here: The dataset and code that supports the finding of this study is openly available at Zenodo with DOI: 10.5281/zenodo.20782185.

## References

[B1] Ackerly D. Knight C. Weiss S. Barton K. Starmer K. (2002). Leaf size, specific leaf area and microhabitat distribution of chaparral woody plants: contrasting patterns in species level and community level analyses. Oecologia 130, 449–457. doi: 10.1007/s004420100805 28547053

[B2] Ackerly D. D. Monson R. K. (2003). Waking the sleeping giant: The evolutionary foundations of plant function. Int. J. Plant Sci. 164, S1–S6. doi: 10.1086/374729 29847380

[B3] Adler P. B. Salguero-Gómez R. Compagnoni A. Hsu J. S. Ray-Mukherjee J. Mbeau-Ache C. . (2014). Functional traits explain variation in plant life history strategies. Proc. Natl. Acad. Sci. 111, 740–747. doi: 10.1073/pnas.1315179111 24379395 PMC3896207

[B4] Alméras T. Fournier M. (2009). Biomechanical design and long-term stability of trees: Morphological and wood traits involved in the balance between weight increase and the gravitropic reaction. J. Theor. Biol. 256, 370–381. doi: 10.1016/j.jtbi.2008.10.011 19013473

[B5] Avalos G. Mulkey S. S. Kitajima K. (1999). Leaf optical properties of trees and lianas in the outer canopy of a tropical dry forest. Biotropica 31, 517–520. doi: 10.1111/j.1744-7429.1999.tb00395.x 40046247

[B6] Barbero M. Loisel R. Quézel P. (1992). Biogeography, ecology and history of Mediterranean Quercus ilex ecosystems. Vegetation 99–100, 19–34. doi: 10.1007/BF00118207 30311153

[B7] Barbosa-Campos M. T. De Castro S. A. B. Kuster V. C. Dos Santos L. N. de Lemos-Filho J. P. Vale F. H. A. (2018). How the long-life span leaves of Ouratea castaneifolia Engl. (Ochnaceae) differ in distinct light conditions. Braz. J. Bot. 41, 403–414. doi: 10.1007/s40415-018-0445-0 30311153

[B8] Beerling D. (1993). The impact of atmospheric CO2 and temperature changes on stomatal density: Observation from Quercus robur Lammas leaves. Ann. Bot. 71, 231–235. doi: 10.1006/anbo.1993.1029 21632986

[B9] Berry J. Bjorkman O. (1980). Photosynthetic response and adaptation to temperature in higher plants. Annu. Rev. Plant Physiol. 31, 491–543. doi: 10.1146/annurev.pp.31.060180.002423 41139587

[B10] Björkman O. (1981). “ Responses to different quantum flux densities,” in Physiological Plant Ecology I. Eds. Lange O. L. Nobel P. S. Osmond C. B. Ziegler H. ( Springer Berlin Heidelberg, Berlin, Heidelberg), 57–107. doi: 10.1007/978-3-642-68090-8_4

[B11] Castro-Díez P. Puyravaud J. P. Cornelissen J. (2000). Leaf structure and anatomy as related to leaf mass per area variation in seedlings of a wide range of woody plant species and types. Oecologia 124, 476–486. doi: 10.1007/PL00008873 28308386

[B12] Chen H. Manning A. K. Dupuis J. (2012). A method of moments estimator for random effect multivariate meta‐analysis. Biometrics 68, 1278–1284. doi: 10.1111/j.1541-0420.2012.01761.x 22551393 PMC4030295

[B13] Chou C. B. Hedin L. O. Pacala S. W. (2018). Functional groups, species and light interact with nutrient limitation during tropical rainforest sapling bottleneck. J. Ecol. 106, 157–167. doi: 10.1111/1365-2745.12823 40046247

[B14] Chow W. S. Melis A. Anderson J. M. (1990). Adjustments of photosystem stoichiometry in chloroplasts improve the quantum efficiency of photosynthesis. Proc. Natl. Acad. Sci. 87, 7502–7506. doi: 10.1073/pnas.87.19.7502 11607105 PMC54775

[B15] De La Riva E. G. Olmo M. Poorter H. Ubera J. L. Villar R. (2016). Leaf mass per area (LMA) and its relationship with leaf structure and anatomy in 34 Mediterranean woody species along a water availability gradient. PLoS One 11, e0148788. doi: 10.1371/journal.pone.0148788 26867213 PMC4750855

[B16] Dieleman W. I. J. Luyssaert S. Rey A. De Angelis P. Barton C. V. Broadmeadow M. S. J. . (2010). Soil [N] modulates soil C cycling in CO_2_ ‐fumigated tree stands: a meta‐analysis. Plant Cell Environ. 33, 2001–2011. doi: 10.1111/j.1365-3040.2010.02201.x 20573048

[B17] Fick S. E. Hijmans R. J. (2017). WorldClim 2: new 1km spatial resolution climate surfaces for global land areas. Int. J. Climatology 37, 4302–4315. doi: 10.1002/joc.5086

[B18] Fonseca C. R. Overton J. M. Collins B. Westoby M. (2000). Shifts in trait‐combinations along rainfall and phosphorus gradients. J. Ecol. 88, 964–977. doi: 10.1046/j.1365-2745.2000.00506.x 37945311

[B19] Freschet G. T. Cornelissen J. H. Van Logtestijn R. S. Aerts R. (2010). Evidence of the ‘plant economics spectrum’ in a subarctic flora. J. Ecol. 98, 362–373. doi: 10.1111/j.1365-2745.2009.01615.x 40046247

[B20] García-Palacios P. Gross N. Gaitán J. Maestre F. T. (2018). Climate mediates the biodiversity–ecosystem stability relationship globally. Proc. Natl. Acad. Sci. 115, 8400–8405. doi: 10.1073/pnas.1800425115 30061405 PMC6099882

[B21] Geber M. A. Griffen L. R. (2003). Inheritance and natural selection on functional traits. Int. J. Plant Sci. 164, 21–42. doi: 10.1086/368233 28788936

[B22] Givnish T. (1988). Adaptation to sun and shade: A whole-plant perspective. Funct. Plant Biol. 15, 63–92. doi: 10.1071/PP9880063 38477348

[B23] Granata M. U. Bracco F. Nola P. Catoni R. (2020). Photosynthetic characteristic and leaf traits variations along a natural light gradient in Acer campestre and Crataegus monogyna. Flora 268, 151626. doi: 10.1016/j.flora.2020.151626 38826717

[B24] Gratani L. (2014). Plant phenotypic plasticity in response to environmental factors. Adv. Bot. 2014, 1–17. doi: 10.1155/2014/208747

[B25] Gratani L. Covone F. Larcher W. (2006). Leaf plasticity in response to light of three evergreen species of the Mediterranean maquis. Trees 20, 549–558. doi: 10.1007/s00468-006-0070-6 30311153

[B26] Guo Q. Q. Li H. E. Gao C. Yang R. (2019). Leaf traits and photosynthetic characteristics of endangered Sinopodophyllum hexandrum (Royle) Ying under different light regimes in Southeastern Tibet Plateau. Photosynthetica 57, 548–555. doi: 10.32615/ps.2019.080

[B27] Guzmán Q. Cordero S. (2013). Growth and photosynthetic performance of five tree seedlings species in response to natural light regimes from the Central Pacific of Costa Rica. Rev. Biol. Trop. 61, 1433–1444. doi: 10.15517/rbt.v61i3.11970 24027934

[B28] Hanson H. C. (1917). Leaf‐structure as related to environment. Am. J. Bot. 4, 533–560. doi: 10.2307/2435253

[B29] Hassan W. U. (2026). Effects of contrasting light on leaf functional traits. Created in BioRender. Available online at: https://BioRender.com/9ju0s2f.

[B30] Hedges L. V. Gurevitch J. Curtis P. S. (1999). The meta-analysis of response ratios in experimental ecology. Ecology 80, 1150–1156. doi: 10.1890/0012-9658(1999)080[1150:TMAORR]2.0.CO;2

[B31] Hill K. E. Hill R. S. Watling J. R. (2015). Do CO2, temperature, rainfall and elevation influence stomatal traits and leaf width in Melaleuca lanceolata across southern Australia? Aust. J. Bot. 62, 666–673. doi: 10.1071/BT14300 38477348

[B32] Hu X. Zhang W. Zhou J. (2016). Plastic responses in tree architecture to different light intensity habitats: A case of Chinese cork oak. Pol. J. Ecol. 64, 500–508. doi: 10.3161/15052249PJE2016.64.4.005

[B33] Jagodziński A. M. Dyderski M. K. Rawlik K. Kątna B. (2016). Seasonal variability of biomass, total leaf area and specific leaf area of forest understory herbs reflects their life strategies. For. Ecol. Manag 374, 71–81. doi: 10.1016/j.foreco.2016.04.050 38826717

[B34] Kikuzawa K. Lechowicz M. J. (2011). Ecology of Leaf Longevity (Tokyo: Springer Tokyo). doi: 10.1007/978-4-431-53918-6

[B35] Klimeš A. Koubek T. Weiser M. Herben T. (2021). Growth plasticity in response to shading as a potential key to the evolution of angiosperm herbs. Plant Ecol. 222, 387–396. doi: 10.1007/s11258-021-01113-9 30311153

[B36] Körner C. (1999). “ Functional plant ecology of high mountain ecosystems,” in Alpine Plant Life Vol. 4 ( Springer, Berlin). doi: 10.1659/mrd.mm265.1

[B37] La Porta N. Bertamini M. Nedunchezhian N. Muthuchelian K. (2006). Photosynthetic changes that occur during aging of cypress (Cupressus sempervirens L.) needles. Photosynthetica 44, 555–560. doi: 10.1007/s11099-006-0071-0 30311153

[B38] Li Y. Liu C. Yang H. Xu L. Wang Q. He N. (2018). Variation in leaf chlorophyll concentration from tropical to cold-temperate forests: Association with gross primary productivity. Ecol. Indic. 85, 383–389. doi: 10.1016/j.ecolind.2017.10.025 38826717

[B39] Liberati A. Altman D. G. Tetzlaff J. Mulrow C. Gøtzsche P. C. Ioannidis J. P. . (2009). The PRISMA statement for reporting systematic reviews and meta-analyses of studies that evaluate health care interventions: Explanation and elaboration. PLoS Med. 6, e1000100. doi: 10.1136/bmj.b2700 19621070 PMC2707010

[B40] Luomala E. Laitinen K. Sutinen S. Kellomäki S. Vapaavuori E. (2005). Stomatal density, anatomy and nutrient concentrations of Scots pine needles are affected by elevated CO_2_ and temperature. Plant Cell Environ. 28, 733–749. doi: 10.1111/j.1365-3040.2005.01319.x 40046247

[B41] Matos F. S. Wolfgramm R. Gonçalves F. V. Cavatte P. C. Ventrella M. C. DaMatta F. M. (2009). Phenotypic plasticity in response to light in the coffee tree. Environ. Exp. Bot. 67, 421–427. doi: 10.1016/j.envexpbot.2009.06.018 38826717

[B42] Mcgill B. Enquist B. Weiher E. Westoby M. (2006). Rebuilding community ecology from functional traits. Trends Ecol. Evol. 21, 178–185. doi: 10.1016/j.tree.2006.02.002 16701083

[B43] McIntyre S. Lavorel S. Landsberg J. Forbes T. D. A. (1999). Disturbance response in vegetation – towards a global perspective on functional traits. J. Veg. Sci. 10, 621–630. doi: 10.2307/3237077

[B44] Miyaji K. Da Silva W. S. Alvim P. D. T. (1997). Productivity of leaves of a tropical tree, Theobroma cacao, grown under shading, in relation to leaf age and light conditions within the canopy. New Phytol. 137, 463–472. doi: 10.1046/j.1469-8137.1997.00841.x 33863072

[B45] Moles A. T. Westoby M. (2000). Do small leaves expand faster than large leaves, and do shorter expansion times reduce herbivore damage? Oikos 90, 517–524. doi: 10.1034/j.1600-0706.2000.900310.x 42325892

[B46] Muraoka H. Tang Y. Koizumi H. Washitani I. (1997). Combined effects of light and water availability on photosynthesis and growth of Arisaema heterophyllum in the forest understory and an open site. Oecologia 112, 26–34. doi: 10.1007/s004420050279 28307371

[B47] Nagata N. Tanaka R. Satoh S. Tanaka A. (2005). Identification of a vinyl reductase gene for chlorophyll synthesis in Arabidopsis thaliana and implications for the evolution of Prochlorococcus species. Plant Cell 17, 233–240. doi: 10.1105/tpc.104.027276 15632054 PMC544501

[B48] Niinemets U. (1997). Acclimation to low irradiance in Picea abies: influences of past and present light climate on foliage structure and function. Tree Physiol. 17, 723–732. doi: 10.1093/treephys/17.11.723 14759897

[B49] Niinemets Ü. (2001). Global-scale climatic controls of leaf dry mass per area, density, and thickness in trees and shrubs. Ecology 82, 453–469. doi: 10.1890/0012-9658(2001)082[0453:GSCCOL]2.0.CO;2

[B50] Niinemets Ü. Keenan T. F. Hallik L. (2015). A worldwide analysis of within‐canopy variations in leaf structural, chemical and physiological traits across plant functional types. New Phytol. 205, 973–993. doi: 10.1111/nph.13096 25318596 PMC5818144

[B51] Niinemets Ü. Tenhunen J. D. (1997). A model separating leaf structural and physiological effects on carbon gain along light gradients for the shade‐tolerant species Acer saccharum. Plant Cell Environ. 20, 845–866. doi: 10.1046/j.1365-3040.1997.d01-133.x 37945311

[B52] Niyogi K. Wolosiuk R. (2015). “ Photosynthesis,” in Biochemistry & Molecular Biology of Plants 2nd ( John Wiley & Sons, Ltd, Chichester, UK), 508–566.

[B53] Oguchi R. Hikosaka K. Hirose T. (2003). Does the photosynthetic light‐acclimation need change in leaf anatomy? Plant Cell Environ. 26, 505–512. doi: 10.1046/j.1365-3040.2003.00981.x 37945311

[B54] Pearcy R. W. (2007). “ Responses of plants to heterogeneous light environments,” in Functional Plant Ecology, 2. Eds. Pugnaire F. Valladares F. (Boca Raton, FL: CRC Press), 213–258. doi: 10.1201/9781420007626-7

[B55] Perot T. Mårell A. Korboulewsky N. Seigner V. Balandier P. (2017). Modeling and predicting solar radiation transmittance in mixed forests at a within-stand scale from tree species basal area. For. Ecol. Manag 390, 127–136. doi: 10.1016/j.foreco.2017.01.023 38826717

[B56] Poorter L. (2001). Light‐dependent changes in biomass allocation and their importance for growth of rain forest tree species. Funct. Ecol. 15, 113–123. doi: 10.1046/j.1365-2435.2001.00503.x 37945311

[B57] Poorter H. Niinemets Ü. Poorter L. Wright I. J. Villar R. (2009). Causes and consequences of variation in leaf mass per area (LMA): a meta‐analysis. New Phytol. 182, 565–588. doi: 10.1111/j.1469-8137.2009.02830.x 19434804

[B58] Poorter H. Niklas K. J. Reich P. B. Oleksyn J. Poot P. Mommer L. (2012). Biomass allocation to leaves, stems and roots: meta‐analyses of interspecific variation and environmental control. New Phytol. 193, 30–50. doi: 10.1111/j.1469-8137.2011.03952.x 22085245

[B59] Poorter H. Pons T. L. Reichgelt T. (2025). Stomatal density and index are more responsive to light intensity than to [CO_2_]: a meta-analysis and implications for paleo-CO_2_ reconstruction. Plant Ecophysiol 1. doi: 10.53941/plantecophys.2025.100001

[B60] Puglielli G. Varone L. Gratani L. Catoni R. (2017). Specific leaf area variations drive acclimation of Cistus salvifolius in different light environments. Photosynthetica 55, 31–40. doi: 10.1007/s11099-016-0235-5 30311153

[B61] Reich P. B. Walters M. B. Ellsworth D. S. (1992). Leaf life‐span in relation to leaf, plant, and stand characteristics among diverse ecosystems. Ecol. Monogr. 62, 365–392. doi: 10.2307/2937116

[B62] Reich P. B. Walters M. B. Ellsworth D. S. (1997). From tropics to tundra: global convergence in plant functioning. Proc. Natl. Acad. Sci. 94, 13730–13734. doi: 10.1073/pnas.94.25.13730 9391094 PMC28374

[B63] Reich P. B. Wright I. J. Ellsworth D. S. (2003). The evolution of plant functional variation: traits, spectra, and strategies. Int. J. Plant Sci. 164, S143–S164. doi: 10.1086/374368

[B64] Rohatgi A. (2024). WebPlotDigitizer (v5.2). software. Available online at: https://automeris.io (Accessed October 16, 2025).

[B65] Scheepens J. F. Frei E. S. Stöcklin J. (2010). Genotypic and environmental variation in specific leaf area in a widespread alpine plant after transplantation to different altitudes. Oecologia 164, 141–150. doi: 10.1007/s00442-010-1650-0 20461412

[B66] Song J. Cao K. Hao Y. Song S. Su W. Liu H. (2019). Hypocotyl elongation is regulated by supplemental blue and red light in cucumber seedling. Gene 707, 117–125. doi: 10.1016/j.gene.2019.04.070 31034942

[B67] Stevenson G. (2015). Plant functional traits associated with frost susceptibility. Lincoln University, New Zealand. BSc Thesis.

[B68] Strain B. Higginbotham K. Mulroy J. C. (1976). Temperature preconditioning and photosynthetic capacity of pinus taeda L. Photosynthetica 10, 47–53.

[B69] Tang H. Dubayah R. (2017). Light-driven growth in Amazon evergreen forests explained by seasonal variations of vertical canopy structure. Proc. Natl. Acad. Sci. 114, 2640–2644. doi: 10.1073/pnas.1616943114 28223505 PMC5347545

[B70] Tucić B. Tomić V. Avramov S. Pemac D. (1998). Testing the adaptive plasticity of Iris pumila leaf traits to natural light conditions using phenotypic selection analysis. Acta Oecologica 19, 473–481. doi: 10.1016/S1146-609X(99)80001-0

[B71] Valladares F. Niinemets Ü. (2007). “ The architecture of plant crowns: from design rules to light capture and performance,” in Functional Plant Ecology, Second Edition. Eds. Valladares F. Pugnaire F. ( CRC Press). doi: 10.1201/9781420007626.ch4

[B72] Vieira T. D. O. Lage-Pinto F. Ribeiro D. R. Dos santos alencar T. Vitória A. P. (2011). Estresse luminoso em plântulas de jequitibá-rosa (Cariniana legalis, Lecythidaceae): monitoramento da capacidade de aclimatação fotossintética sob duas intensidades de luz. Rev. Vértices 13, 129–142. doi: 10.5935/1809-2667.20110029

[B73] Violle C. Vile D. Vile D. Kazakou E. Fortunel C. Hummel I. . (2007). Let the concept of trait be functional! Oikos 116, 882–892. doi: 10.1111/j.0030-1299.2007.15559.x 40046247

[B74] Walters R. G. (2004). Towards an understanding of photosynthetic acclimation. J. Exp. Bot. 56, 435–447. doi: 10.1093/jxb/eri060 15642715

[B75] Woodward F. I. (1983). The significance of interspecific differences in specific leaf area to the growth of selected herbaceous species from different altitudes. New Phytol. 95, 313–323. doi: 10.1111/j.1469-8137.1983.tb03498.x 40046247

[B76] Wright I. J. Reich P. B. Cornelissen J. H. Falster D. S. Garnier E. Hikosaka K. . (2005). Assessing the generality of global leaf trait relationships. New Phytol. 166, 485–496. doi: 10.1111/j.1469-8137.2005.01349.x 15819912

[B77] Wright I. J. Reich P. B. Westoby M. Ackerly D. D. Baruch Z. Bongers F. . (2004). The worldwide leaf economics spectrum. Nature 428, 821–827. doi: 10.1038/nature02403 15103368

[B78] Wu C. Niu Z. Tang Q. Huang W. (2008). Estimating chlorophyll content from hyperspectral vegetation indices: modeling and validation. Agric. For. Meteorol. 148, 1230–1241. doi: 10.1016/j.agrformet.2008.03.005 38826717

[B79] Wu T. Wu S. Wu S. Cheng M. Qi L. Shao G. . (2023). Retrieving rice (Oryza sativa L.) net photosynthetic rate from UAV multispectral images based on machine learning methods. Front. Plant Sci. 13, 1088499. doi: 10.3389/fpls.2022.1088499 36762179 PMC9905687

[B80] Xu W. Zhang B. Xu Q. Gao D. Zuo H. Ren R. . (2024). Enhanced carbon storage in mixed coniferous and broadleaf forest compared to pure forest in the north subtropical–warm temperate transition zone of China. Forests 15, 1520. doi: 10.3390/f15091520 30654563

[B81] Yan W. Zhong Y. Shangguan Z. (2017). Contrasting responses of leaf stomatal characteristics to climate change: a considerable challenge to predict carbon and water cycles. Glob. Change Biol. 23, 3781–3793. doi: 10.1111/gcb.13654 28181733

[B82] Yang F. Feng L. Liu Q. Wu X. Fan Y. Raza M. A. . (2018). Effect of interactions between light intensity and red-to- far-red ratio on the photosynthesis of soybean leaves under shade condition. Environ. Exp. Bot. 150, 79–87. doi: 10.1016/j.envexpbot.2018.03.008 38826717

[B83] Yang Y. Wang H. Harrison S. P. Prentice I. C. Wright I. J. Peng C. . (2019). Quantifying leaf‐trait covariation and its controls across climates and biomes. New Phytol. 221, 155–168. doi: 10.1111/nph.15422 30272817

[B84] Zhang Y. Guo Q. Luo S. Pan J. Yao S. Gao C. . (2022). Leaf morphology, structure, and function of Camellia oleifera (Abel) under different light regimes. Eur. J. Hortic. Sci. 87, 1–7. doi: 10.17660/eJHS.2022/036 42372210

[B85] Zhang K. L. Leng Y. N. Hao R. R. Zhang W. Y. Li H. F. Chen M. X. . (2024). Adaptation of high-altitude plants to harsh environments: application of phenotypic-variation-related methods and multi-omics techniques. Int. J. Mol. Sci. 25, 12666. doi: 10.3390/ijms252312666 39684378 PMC11641277

[B86] Zhang X. Sun Y. Landis J. B. Shen J. Zhang H. Kuang T. . (2021). Transcriptomes of Saussurea (Asteraceae) provide insights into high-altitude adaptation. Plants 10, 1715. doi: 10.3390/plants10081715 34451759 PMC8402177

[B87] Zhi-Zhen L. Dong-Huan L. Zhao S. W. Jiang C. D. Shi L. (2014). Mechanisms of photoinhibition induced by high light in Hosta grown outdoors. Chin. J. Plant Ecol. 38, 720–728. doi: 10.3724/SP.J.1258.2014.00067

