# Supplementary material for: Unravelling the role of contrasting light environments in shaping leaf functional traits: a global meta-analysis
Source: Front Plant Sci. 2026 Jul 14;17:1884395. doi: 10.3389/fpls.2026.1884395 (PMC13407534; doi:10.3389/fpls.2026.1884395)
Supplement: Supplementary file 1 [file DataSheet1.docx]

**Supplementary Material**

**Unravelling the role of contrasting light environments in shaping leaf functional traits: a global meta-analysis**

Wajee ul Hassan^1^, Xiping Cheng^*2^, Yanfan Wang^2^, Runze Li^2^, Pengyue Dai^2^, Jing Chen^3^
^1^College of Forestry, Southwest Forestry University, Kunming 650224, Yunnan, China

^2^College of Soil and Water Conservation, Southwest Forestry University, Kunming 650224, Yunnan, China

^3^College of Gardening and Horticulture, Southwest Forestry University, Kunming 650224, Yunnan, China;

**Corresponding author**: Xiping Cheng

Email: xipingcheng@swfu.edu.cn

Tel: +86 13629427426

**Table S1.** Publication bias test for different response variables under contrasting light environments

| Response variables | N | 5N + 10 | Fail-safe N |
| --- | --- | --- | --- |
| SLA | 200 | 1010 | 643723 |
| LMA | 45 | 235 | 240049 |
| Pn | 104 | 525 | 48823 |
| Chlorophyll Content | 110 | 560 | 117842 |
| Stomatal Density | 53 | 275 | 25003 |
| LDMC | 24 | 125 | 147 |

N is the number of observations for comparison. The results were considered credible when Fail-safe N > 5N + 10.

**
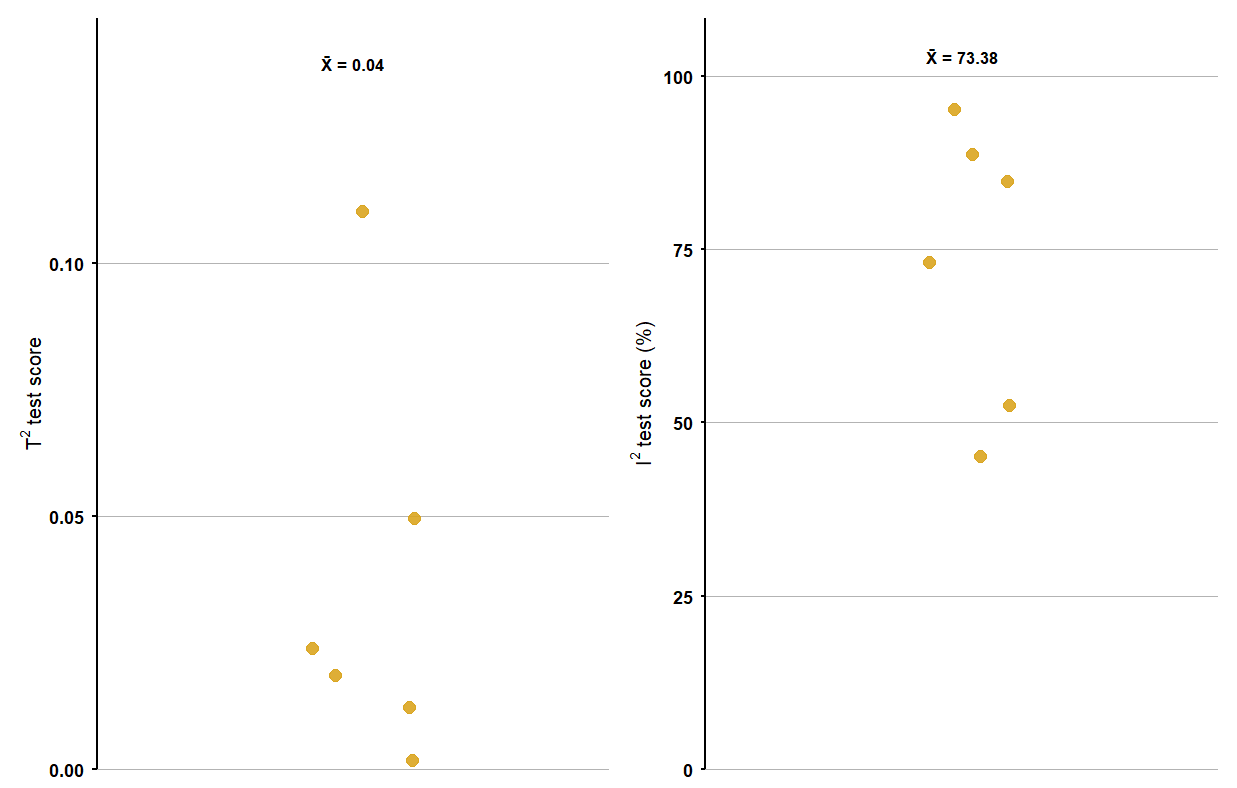
**

**Figure S1: The I^2^ and T^2^ heterogeneity tests for six response variables under the contrasting light environments.**
